# That “Memorial Meeting”

**Published:** 1890-01

**Authors:** 


### THAT "MEMORIAL MEETING."

The meeting proposed by Dr. Allport, and first published by this Journal in an editorial on the American Dental Association, in the October number, for an anniversary meeting in that society upon the occasion of the World's Fair, in 1892, seems to meet with favor in several quarters. The December *Cosmos* has a comprehensive editorial on the subject, in which the position taken by us throughout the discussion on the Second International Dental Congress is thoroughly sustained. It says,—

"It seems, therefore, eminently fit and proper that the American Dental Association should assume the direction of a dental branch of the celebration of the birthday of America, and we earnestly hope that the youngest of the professions and one by birthright distinctively American may look with confidence to the American Dental Association for an arrangement providing for a fitting celebration in 1892."

That is what we have claimed from the first,—that, in the event of a dental congress, it should be under the supervision of the American Dental Association, and that any and all benefits to be derived from such a meeting should accrue to the already existing organizations in America. It matters not whether the World's Fair be held in New York, Chicago, or Washington, all true lovers of our profession should join heartily and help make the movement a success. The matter should be fully discussed before the next meeting of the association, and some tangible plan be ready for presentation at that time, so that committees could be appointed in order to insure arrangements that shall be in keeping with the event to be celebrated.

It has been remarked that the title "Memorial Meeting" is hardly appropriate, and it has even been hinted that the name suggests a death in the family, and that it is necessary to hold a memorial meeting for the American Dental Association. Such is not the case. It is not a memorial meeting *for* the American Dental Association but *in* the American Dental Association for the late Second International Dental Congress, which suffered an untimely death at Saratoga last summer.

